# The moderating effect of employee growth climate on the relationship between work engagement and job outcomes among plantation workers in North Sumatra, Indonesia

**DOI:** 10.3389/fpsyg.2022.968572

**Published:** 2022-11-28

**Authors:** Vivi Gusrini Rahmadani, Wilmar B. Schaufeli, Rahma Fauzia, Dina Nazriani

**Affiliations:** ^1^Faculty of Psychology, Universitas Sumatera Utara, Medan, Indonesia; ^2^Occupational and Organisational Psychology and Professional Learning, Faculty of Psychology and Educational Sciences, KU Leuven, Leuven, Belgium

**Keywords:** employee growth climate, work engagement, job outcomes, intra-role behavior, employee learning, innovative work behavior, plantation workers, agricultural industry

## Abstract

The current study investigates the moderating effect of employee growth climate on the relationship between work engagement and job outcomes among plantation workers in North Sumatra, Indonesia. Three individual-level job outcomes are investigated, namely, intra-role behavior, employee learning, and innovative work behavior. Six hundred and seven Indonesian plantation workers from one of the biggest palm oil plantations in Indonesia participated. Work engagement and employee growth climate were positively related to the three types of job outcomes, as expected. Furthermore, the relationship between work engagement-intra-role behavior and work engagement-innovative work behavior was moderated by employee growth climate. However, no moderating effect of employee growth climate was observed for the relationship between work engagement-employee learning. Thus, organizations may create programs to foster employee growth climate and aware of their employees’ learning behaviors.

## Introduction

One of the priority goals in organizations could be how to assess and nurture work engagement among their employees, as it has a positive impact on employees’ job outcomes at the individual, team, and organizational levels (Sartori and Ceschi, 2013). Furthermore, job outcomes are multidimensional that must be specified because organizations may focus on certain targeted performances or outcomes (Sartori et al., 2022). In the current study, job outcomes are investigated with three different variables, namely, intra-role behavior, employee learning, and innovative work behavior. Previous research showed that work engagement has a significant and positive influence on those three job outcomes; however, we try to explain which context actually increases each of the job outcomes of the engaged workers. The context used in this study is called employee growth climate. Building from the relationship between job resources and work engagement, Schaufeli (2016) introduces employee growth climate as organizational policies, practices, and procedures that encourage an employee’s personal and professional growth. Thus, we try to measure this specific context of organizational climate that is needed to foster work engagement.

This study makes two contributions to the literature. First, by explaining the moderating role of employee growth climate on the relationship between work engagement and job outcomes, this study gives insight into the context in which work engagement impacts certain individual job outcomes. Second, research to date provides scarce information on engagement in an Indonesian-Asian context with the agricultural industry, that is, a labor-intensive sector with less advancement in technology; this stresses the need for sound human resources management.

Indonesia, with its enormous agricultural and plantation area, particularly in North Sumatra, is renowned as an agrarian country with a developed and growing agro-industry. Agro-industry, specifically plantations, is one of the industrial sectors that contribute significantly to the national economy, in terms of both national income and job creation. However, continual technological advancements, dynamic and unexpected social and political upheavals, and pandemic circumstances that have afflicted the entire world, including Indonesia, are the primary impediments to strengthening the agro-industry sector (Ansari, 2021). Despite numerous obstacles, agribusiness has enormous potential to contribute significantly to the progress and change of economic growth (Setiawan, 2021). In terms of developing the national agro-industry in the VUCA (Volatility, Uncertainty, Complexity, and Ambiguity) era, the government encourages industries to take active steps that include ensuring the achievement of goals and targets and continuing to foster the spirit of innovation and improvement, so that businesses are always prepared to anticipate the worst-case scenario (Ansari, 2021). The role of human resources, the plantation workers, that is viewed as critical and the strategic objective of increasing the industrial sector’s competitiveness and productivity, has been achieved in terms of the contribution of agricultural exports to national exports, but not in terms of human resources (HR) productivity (Nursyamsi, 2020). The current study has been carried out in North Sumatra, Indonesia to portray the role of organizational climate, specifically employee growth climate, on the relationship between work engagement and intra-role behavior, employee learning, and innovative work behavior of workers. The current study focuses on moderating processes in which employee growth climate is assumed to play an important role.

The study examines job outcomes through the lens of three dimensions: job performance, employee learning, and innovative work behavior. Job performance is defined as employees’ behavior that formally conforms to the objectives of their employment (intra-role behavior) (Goodman and Svyantek, 1999). Additionally, given the uncertain dynamics of the business world, employees are expected to continuously learn or adapt to the influence of external pressure on their activities, and thus to have a high level of employee learning. In an organization, a worker is said to be learning if he is constantly inquiring when something is wrong with the way he is performing his tasks, seeking information, experimenting, talking, and getting feedback whenever an action is performed to resolve his work difficulties (Edmondson, 1999). When employees are content with performing repetitive duties, adaptation occurs very slowly; businesses fall behind and are less equipped to innovate or transform to meet the demands of a fast-changing business world (Pohan, 2011). Additionally, the company’s aspirations for innovation will necessitate workers who exhibit innovative behavior. Innovative work behaviors are those that demonstrate the ability to generate, promote, and implement new ideas (Janssen, 2000).

Both work engagement and organizational climate have demonstrated increasing job performance. Employees’ perceptions of organizational climate have been demonstrated to influence job performance (Parker et al., 2003), learning (Chahar et al., 2019), creativity (Hsu and Fan, 2010; Wang and Rode, 2010), and creative work behavior (Wang and Rode, 2010; Letchumanasamy and Ramudu, 2014). A positive organizational environment is also a resource for the organization, comprising physical, social, and organizational aspects at work. Moreover, positive work attitudes, psychological well-being, and job performance have been achieved by stimulating work engagement among employees (Salanova and Schaufeli, 2008; Halbesleben, 2010; Schaufeli, 2012). Furthermore, engaged employees exhibit innovative work behaviors in the workplace (Chang et al., 2013). Finally, a high level of work engagement boosts employees’ personal initiative and innovativeness (Hakanen et al., 2008; Salanova and Schaufeli, 2008).

### Hypothesis development

In the current study, three critical job outcomes are measured to ensure an organization’s survival and competitiveness: intra-role behavior, employee learning, and innovative work behavior at the individual level. Organizational studies show a positive correlation between these three outcomes (Jiménez-Jiménez and Sanz-Valle, 2011; Moreno et al., 2013; Rahmadani et al., 2020). Intra-role behavior is often interchangeably evaluated as job performance, which sometimes includes both intra-role and extra-role behavior. In this study, we focus on intra-role behavior. Task and contextual performances are related to intra-role behavior, which is represented by the formal job description or key performance indicators (Pohan, 2011). The behaviors performed by the employees are typically the same as the activities assigned to them by the organization. Employees who achieve the goals of assigned tasks labeled as performed, to be more specific, demonstrate expertise in their formal assigned work-related tasks. As the business world is continuously changing, organizations are challenged and must learn to adapt. At the individual level, employee learning is inevitable. Employee learning is viewed as a continuous process of reflection and action characterized by questions, feedback, experimentation, reflection on results, and discussion of errors or unexpected consequences of actions (Edmondson, 1999). Rather than viewing learning as a one-time event, employees are encouraged to view it as a perpetual process that may include future assignments and career development as well (McCauley and Hezlett, 2001). Similar to learning, innovativeness is a prerequisite for organizations to sustain and be competitive. At the individual level, organizations encourage their employees to perform innovative behavior. Innovative work behavior is defined as complex behavior that consists of three distinct behavioral tasks: idea generation, idea promotion, and idea realization (Janssen, 2000). When intra-role behavior, employee learning, and innovative work behavior are performed, the competitive value of human resources is increased, which supports organizations in achieving their goals. However, while these outcomes can be obtained with the support of employees’ positive attributes at work, in this study, we focus on work engagement.

Work engagement, also called employee engagement, has become a prominent topic in business and academia due to the positive impact it has on both employees and the organizations for which they work. Gallup, a management consulting firm, introduced it in the 1980s (Wah, 1999). Later, Kahn (1990) popularized the concept in academia, defining engaged employees as those who express themselves physically, cognitively, emotionally, and mentally while performing their assigned tasks (Kahn, 1990). Thus, when employees are engaged, they bring all aspects of themselves to their performance – cognitive, emotional, and physical. In their seminal, synthesized paper, Macey and Schneider (2008) define engagement as “a desirable condition [that] has an organizational purpose and entails involvement, commitment, passion, enthusiasm, focused effort, and energy” (p. 4). However, this definition has been criticized for being overly broad and serving as a catch-all for other, related concepts (Saks and Gruman, 2014). Similarly, Christian et al. (2011) defined engagement as a broad concept that “encompasses a holistic investment of one’s cognitive, emotional, and physical energies” (p. 97) (Christian et al., 2011). By contrast, Schaufeli et al. (2002) defined engagement as “a positive, fulfilling, work-related state of mind characterized by vitality, dedication, and absorption” (p. 74). Work engagement is defined here as a distinct concept (Schaufeli, 2013).

Vigor is higher energy and perseverance, enthusiasm, inspiration, and involvement in a task. Schaufeli (2013) contended that work engagement is the association an employee has with their jobs, while employee engagement also includes the relationship between employees and the organization. This definition contradicts previous broad definitions of employee engagement. The difference between engagement and other well-known concepts such as extra-role behaviors and organizational commitment is unclear because they include relationships with the organization. The Utrecht Work Engagement Scale (UWES) is a concise, sound, and consistent questionnaire that defines work engagement as a mixture of vigor, enthusiasm, and engagement (Schaufeli, 2012; Schaufeli, 2013). Over 80% of academic research on engagement uses the UWES (Bailey et al., 2017). Engaged employees boost organizational performance at individual and team levels. The improved performance is also observed at organizational and departmental levels. Engaged employees are loyal and carry out their job responsibilities with enthusiasm (Markos and Sridevi, 2010). They are intrinsically motivated, proactive, creative, and more healthy and committed to the organization (Huhtala and Parzefall, 2007; Salanova and Schaufeli, 2008; Halbesleben, 2010; Schaufeli, 2012). Furthermore, work engagement is critical in maintaining a competitive edge (Bakker et al., 2008). It enhances labor productivity, reduces employee turnover, increases customer satisfaction, job satisfaction, and profitability. Besides, like Chang et al. (2013) argued that engaged employees show innovative behaviors in their work. They are also more creative than employees who are less engaged (Demerouti et al., 2015). Besides, various scholars have associated high levels of work engagement with high financial returns, high service quality, workplace safety, superior business unit performance, and business growth (Harter et al., 2002; Salanova et al., 2005; Xanthopoulou et al., 2009; Gorgievski and Bakker, 2010; Nahrgang et al., 2011). Similarly, work engagement is positively related to better performance results and job attitudes such job involvement and satisfaction. Therefore, work engagement is important to both employees and organizations for which they work (Christian et al., 2011).

Schaufeli and Bakker (2010) established a Job Demands-Resources (JD-R) model, incorporating work engagement (Schaufeli and Bakker, 2010). The model perceives work engagement as a mediator between the influence of job resources and individual resources on organizational and personal outcomes, whereas in contrast, burn out mediates between job demands and negative outcomes (health problems) (Schaufeli and Taris, 2014). The former is called the motivational process, whereas the latter is called the health impairment process. The current study focuses exclusively on the motivational process of the JDR-model. As outlined by Demerouti et al. (2001), JD-R model defines job resources as the physical, social, or organizational aspects of a job that can accomplish work objectives, decrease job demands and related physiological and psychological costs, and promote personal growth and development. According to Hackman and Oldham (1980), job resources have an intrinsic motivational impact and are essential for work engagement.

One of the organizational aspects that promote employees’ growth and development is organizational climate. Organizational climate is the shared perception by employees and the meaning they associate with workplace practices, procedures, policies, and behaviors that the organization support, reward, and expect (Schneider et al., 2017). The current study investigates employee growth climate in line with Schneider’s (1975) argument that the features of an organizational climate depend on two climates, namely overwork and employee growth climates. The current study focused on the effect of employee growth climate on the relationship between work engagement and job outcomes (Schaufeli, 2015). Organizational climate is linked to significant work investment, namely employee growth climate. A high-performance climate enhances engagement, resulting in better business outcomes (Thompson, 2010).

Previous research studied the influence psychological safety climate on work engagement in educational institutions (Dollard and Bakker, 2010). The study reveals that psychological safety climate foresees the level to which teachers are engaged after 1 year of job resource increase. The current study adopts a similar approach, however, it focuses on employee growth climate, including organizational practices, policies, and procedures that encourage employee professional growth and development. Employee growth climate is linked to the availability of job resources whereby job resources enhance employee engagement. Various scholarly papers have documented how job resources such as performance feedback, social support, job control, learning and career opportunities, enhance employee engagement (Schaufeli and Taris, 2014). Therefore, it can be argued that organizational practices, policies, and procedures influence employee personal and professional growth, resulting in more employee engagement due to increased availability of job resources (Schaufeli, 2015). When an organization views employee growth as important, feedback practices reinforce learning and innovation. Organizational policies that stimulate employees’ careers provide a conducive and safe work environment focusing on the strength of each employee that is employee growth climate.

**Figure 1:**
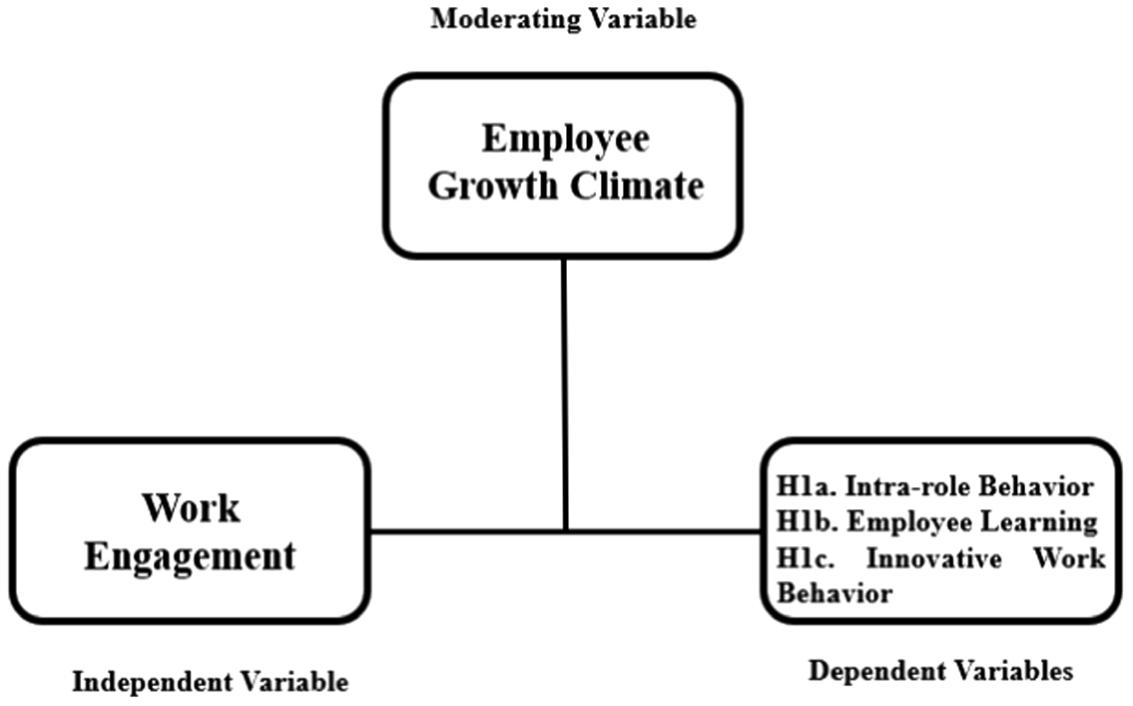
Moderating effect of employee growth climate on the relationship between work engagement and job outcomes: intra-role behavior (Hypothesis 1), employee learning (Hypothesis 2), innovative work behavior (Hypothesis 3).

In the current study, we expect that the relationship between work engagement and job outcomes will differ with different levels of employee growth climate, as perceived by employees. To the best of our knowledge, no study exists on the role that work engagement and a specific type of organizational climate, namely, employee growth climate, play in promoting certain job outcomes. However, some studies show the importance of a positive organizational climate for stimulating job outcomes (Luthans et al., 2008). Employee growth climate can also be seen as a positive organizational climate that provides employees with job resources and thus, from the perspective of Conservation Resources Theory (Hobfoll, 2002; Ng and Feldman, 2012), enables employees to achieve positive job outcomes, namely, intra-role behavior, employee learning, and innovative work behavior. Engaged workers will show a discretionary effort to achieve their goals in work; intra-role behavior. The higher the organizational climate that facilitates employees to obtain the goals by appreciating them when they take on responsibility and work, the more strongly the engaged workers will fulfill their work goals, namely, intra-role behavior. In a similar vein, employee growth climate also stimulates employees to learn and develop themselves; employees are seen as an important part of the organization, and the relationship between the level of employees’ work engagement and employee learning will be stronger. Finally, employee growth climate encourages creative thinking and new solutions from employees. Thus, this kind of climate will strengthen the connection of engaged workers to performing innovative work behaviors. Figure 1 displays the research model of this study.

#### Hypothesis

Thus, we formulate:

*H1*: Employee growth climate moderates the relationship between work engagement and intra-role behavior in the sense that higher levels of perceived employee growth climate strengthen this relationship.

*H2*: Employee growth climate moderates the relationship between work engagement and employee learning in the sense that higher levels of perceived employee growth climate strengthen this relationship.

*H3*: Employee growth climate moderates the relationship between work engagement and innovative work behavior in the sense that higher levels of perceived employee growth climate strengthen this relationship.

## Materials and methods

This is a quantitative correlational survey research with a cross-sectional design. Using 5 study variables namely work engagement as the independent variable, employee growth climate as the moderator variable, and three dependent variables of intra-role behavior, employee leaning and innovative work behavior, the research model was empirically tested in Indonesia.

### Sample and procedure

Conveniently selected, we tested our research model in one of the biggest palm oil plantations in Indonesia. After the official agreement with the company, 607 workers were participated in this study. 611 employees returned the survey out of 700 targeted participants (response rate 87.3%; four surveys could not be used for further analyses because they were not filled out completely). All of the participants worked at plantation sites among various regions in North Sumatra. All participants were men; their mean age was 44.6 years (SD = 7.7); 23.2% had completed elementary education, 59.6% had completed secondary education, 0.2% had completed professional higher education, 16.5% had completed a bachelor’s degree, and 0.5% had completed a master’s degree; more than half of the participants (56.5%) had over 20 years of job tenure. The surveys were distributed to participants in sealed envelopes. Participants were given a written description of the study and asked to consent to participate by signing the attached form. The surveys were completed anonymously during the participants’ normal working hours and were returned in a sealed envelope to the Human Resources Department collectively per unit within a maximum of 2 weeks. The study was conducted on a voluntary basis, and all responses were kept confidential. No ethical code was violated. The entire data collection process took 3 months.

### Measurements

A booklet, consisting of five summated-rating questionnaires, was used to measure work engagement, intra-role behavior, employee learning, innovative work behavior, and employee growth climate. All items in the questionnaires were translated from English into Bahasa Indonesia following the double translation procedure (Brislin, 1970).

#### Intra-role behavior

Individual job performance, namely, intra-role behavior, was assessed using four items from the Job Performance Scale (Goodman and Svyantek, 1999). The sample item is “I demonstrate expertise in all job-related tasks.” Cronbach’s alpha value for this scale were 0.75.

#### Employee learning

An employee learning questionnaire, consisting of six items from a seven-item scale, was used to measure learning behavior (Edmondson, 1999). In this study, to transform the original questionnaire that measures team learning, the referent of all items was changed from “My team” to “I.” Item 2 (“I tend to handle differences of opinion privately or off-line, rather than addressing them directly as a group”) has a very low factor loading according to our CFA, and we removed it for cultural reasons. Indonesians used to maintain harmony in their community and prevent direct confrontation when dealing with conflicts. The sample item of the employee learning questionnaire included the item “I frequently seek new information that leads me to make important changes.” Cronbach’s alpha value for this scale was 0.77, whereas when the original seven items were used, it was 0.74.

#### Innovative work behavior

The four-item innovative work behavior questionnaire was used to assess innovative work behavior (Janssen, 2000). Three components were involved: idea generation, idea promotion, and idea application, with sample items such as “I create new ideas for difficult issues,” “I make important organizational members enthusiastic for innovative ideas,” and “I introduce innovative ideas into the work environment in a systematic way.” Cronbach’s alpha value for this scale were 0.92.

#### Work engagement

The Utrecht Work Engagement Scale (UWES) was used to measure work engagement (Schaufeli et al., 2006). It consisted of nine items from three components: vigor, dedication, and absorption. As proven by various studies in various countries, this scale has satisfactory psychometric properties. Sample items are: “At my job, I feel strong and vigorous” (vigor), “My job inspires me” (dedication), and “I am immersed in my work” (absorption). The value of Cronbach’s alpha for this scale were 0.87.

#### Employee growth climate

Six out of eight items of the employee growth climate questionnaire developed by Schaufeli (2016) were used. The plantation workers evaluated the extent to which their organization encouraged their growth and development. Item 4 and 7 were deleted because they were in a separate factor from the rest six items (“At my workplace, it does not matter to make mistakes because it contributes to learning”; “At my workplace, it is not necessary to work according to the book”). We concluded that these items were not applied to company’s policy and procedure perceived by the employee. The sample of items included “In the organization where I work, employees are encouraged to come up with new ideas and solutions.” All items were measured on a rating scale ranging from 1 (strongly disagree) to 5 (strongly agree). Cronbach’s alpha values for this scale were 0.79, whereas when the original eight items were used, the values went down to 0.72.

## Results

### Descriptive statistics

Data was analyzed using IBM SPSS Statistics. Table 1 presents the mean scores, standard deviations, Cronbach’s alpha, and correlations between the study variables. The result show a positive relationship between work engagement and all job outcomes. The current study investigates job outcomes with three different variables, namely, intra-role behavior, employee learning, and innovative work behavior. The Cronbach’s alpha reliability coefficients in this study were higher than 0.7, thus proving the reliability of the measurements. In sequence, work engagement has the highest mean score (*M* = 4.34), followed by employee growth climate (*M* = 4.17), and intra-role behavior (*M* = 3.92), with the employee learning and innovative behavior variable having the lowest mean score (*M* = 3.42). The correlations between all variables are shown in Table 1. Work engagement shows the strongest relation with intra-role behavior (*r* = 0.41), followed by innovative work behavior (*r* = 0.36) and growth climate (*r* = 0.31). The lowest level of relationship was found between work engagement and employee learning (*r* = 0.28).

**Table 1:** Means (*M*), standard deviations (SD), Cronbach’s α and correlation coefficients of the study variables (*N* = 607).

Variables	*M*	SD	*α*	1	2	3	4	5
1. Work engagement	4.34	0.57	0.87					
2. Intra-role behavior	3.92	0.58	0.75	0.41^**^				
3. Employee learning	3.42	0.71	0.77	0.28^**^	0.39^**^			
4. Innovative work behavior	3.42	0.83	0.92	0.36^**^	0.59^**^	0.59^**^		
5. Employee growth climate	4.17	0.45	0.79	0.31^**^	0.27^**^	0.29^**^	0.28^**^	

**p < 0.01.

### Testing of hypotheses

Three hypotheses were tested in this study. The first hypothesis stated that employee growth climate has a moderating effect between work engagement and intra-role behavior. The second hypothesis stated that employee growth climate moderates the relationship between work engagement and employee learning. Finally, the third hypothesis stated that employee growth climate moderates the relationship between work engagement and innovative work behavior. To test these hypotheses, analyses were carried out with the PROCESS Macro modeling tools in the SPSS statistical calculation program.

Model 1 of SPSS PROCESS Macro (Hayes, 2017) was used to investigate the moderating effect of employee growth climate on the relationship between work engagement and job outcomes. Further results show that employee growth climate significantly moderates only the relationship between work engagement, intra-role, and innovative work behavior. The relationship between employee learning and work engagement is not moderated by employee growth climate. Table 2 shows that employee growth climate has a significant and positive moderating effect on the relationship between intra-role behavior and work engagement (hypothesis 1 confirmed). Likewise, Table 3 shows that employee growth climate has a significant moderating effect on the relationship between innovative work and work engagement (hypothesis 3 confirmed). Table 4, however, shows that employee growth climate does *not* have a significant moderating effect on the relationship between employee learning and work engagement, thus, hypothesis 2 is not supported.

**Table 2:** The moderation of employee growth climate on the relationship between work engagement and intra-role behavior (*N* = 607).

	B	SE	*t*	*p*
Constant	16.99	5.72	2.97	0.00^**^
Employee growth climate	−0.32	0.24	−1.37	0.17
Work engagement	−0.08	0.46	−0.18	0.86
Employee growth climate ^*^Work Engagement	0.01	0.00	1.94	0.05^**^

R^2^ = 0.20. F(3,603) = 49.18.

*p < 0.05; **p < 0.01.

**Table 3:** The moderation of employee growth climate on the relationship between work engagement and innovative work behavior (*N* = 607).

	B	SE	*t*	*p*
Constant	53.18	18.77	2.83	0.00^*^
Employee growth climate	−1.67	0.78	−2.15	0.03^*^
Work engagement	−0.91	0.48	−1.89	0.05^*^
Employee growth climate ^*^ Work engagement	0.06	0.02	2.84	0.00^**^

R^2^ = 0.17. F(3,603) = 42.53.

*p < 0.05; **p < 0.01.

**Table 4:** The moderation of employee growth climate on the relationship between work engagement and employee learning (*N* = 607).

	B	SE	*t*	*p*
Constant	15.38	11.1	1.38	0.17
Employee growth climate	−0.09	0.28	−0.33	0.74
Work engagement	−0.08	0.46	−0.18	0.86
Employee growth climate ^*^ Work engagement	0.01	0.01	0.95	0.34

R^2^=0.12. F(3,603) = 27.99.

*p < 0.05; **p < 0.01.

#### The nature of the moderation

**Figure 2:**
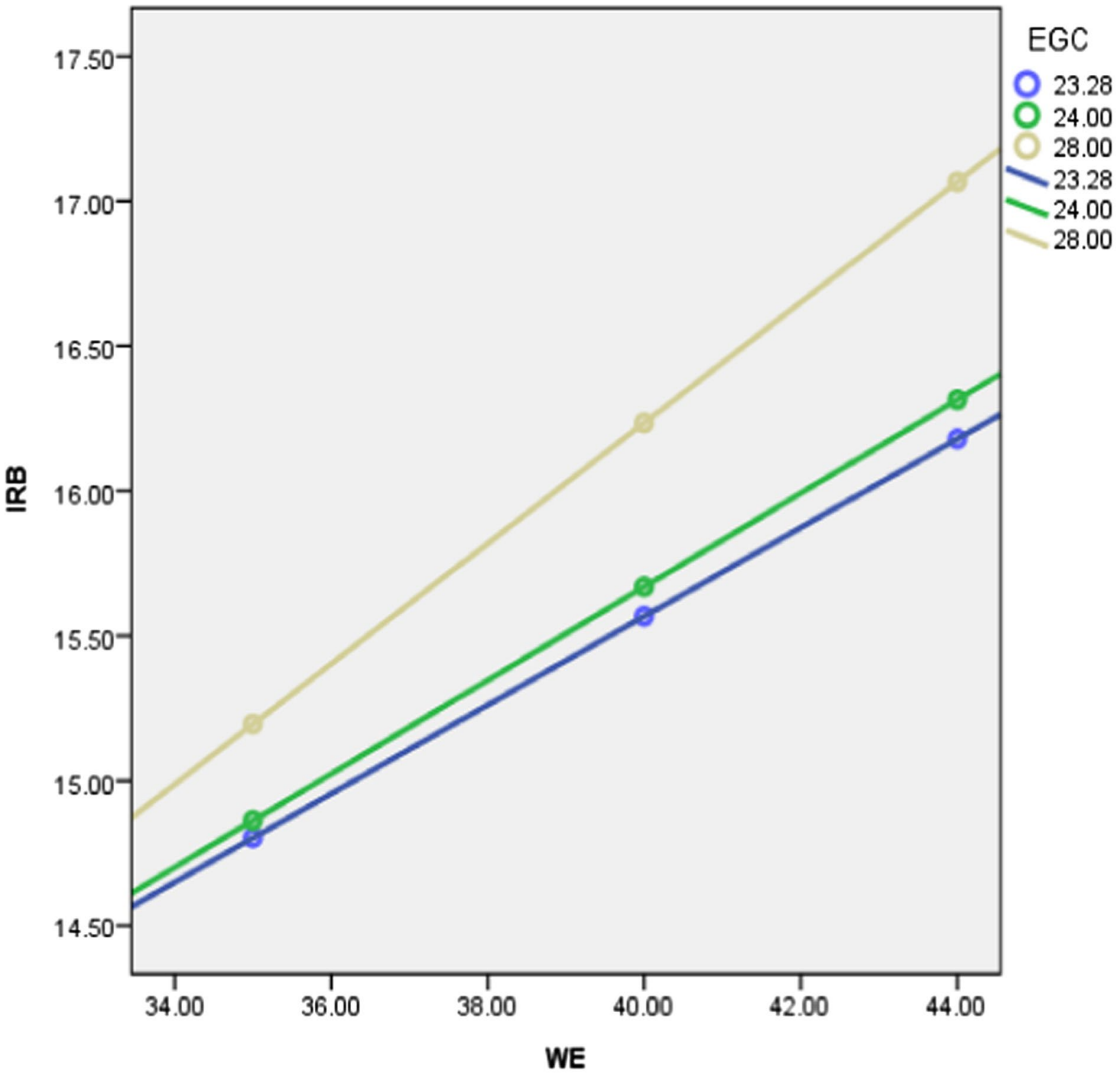
Interaction plot of intra-role behavior as a function of work engagement for low (−1SD), average, and high (+1SD) levels of employee growth climate.

As shown in Table 2, the main effect of employee growth climate and work engagement on intra-role behavior are not significant. However, and more importantly, the moderating effect of employee growth climate on the relationship between intra-role behavior and work engagement is significant [*b* = −0.11, *t*(603) = −0.81, *p =* 0.42]. Based on this analysis, hypothesis 1 is accepted. We then computed separate regression lines for high employee growth climate as perceived by the employee (1 SD above the mean), average employee growth climate, and low employee growth climate (1 SD below the mean), and plotted this (see Figure 2).

Figure 2 displays the interaction between work engagement in the prediction of intra-role behavior. The figure illustrates that employee growth climate moderates the relationship between work engagement and intra-role behavior in such a way that work engagement is positively associated with intra-role behavior for high, average, and low employee growth climate. This finding supports hypothesis 2a. Hence, it is concluded that the relationship between work engagement and is moderated by employee growth climate.

A similar moderation analyses was carried out for employee learning and innovative work behavior. Table 4 shows the results of the moderating effect of employee growth climate on the relationship between work engagement and employee learning [*b* = −0.09, *t*(603) = −0.33, *p* = 0.74]. In this analysis, the result of the interaction shows a non-significant moderating effect, resulting in the rejection of hypothesis 2. Thus, it is concluded that the relationship between work engagement and employee learning is not moderated by employee growth climate (Hypothesis 2b not supported).

**Figure 3:**
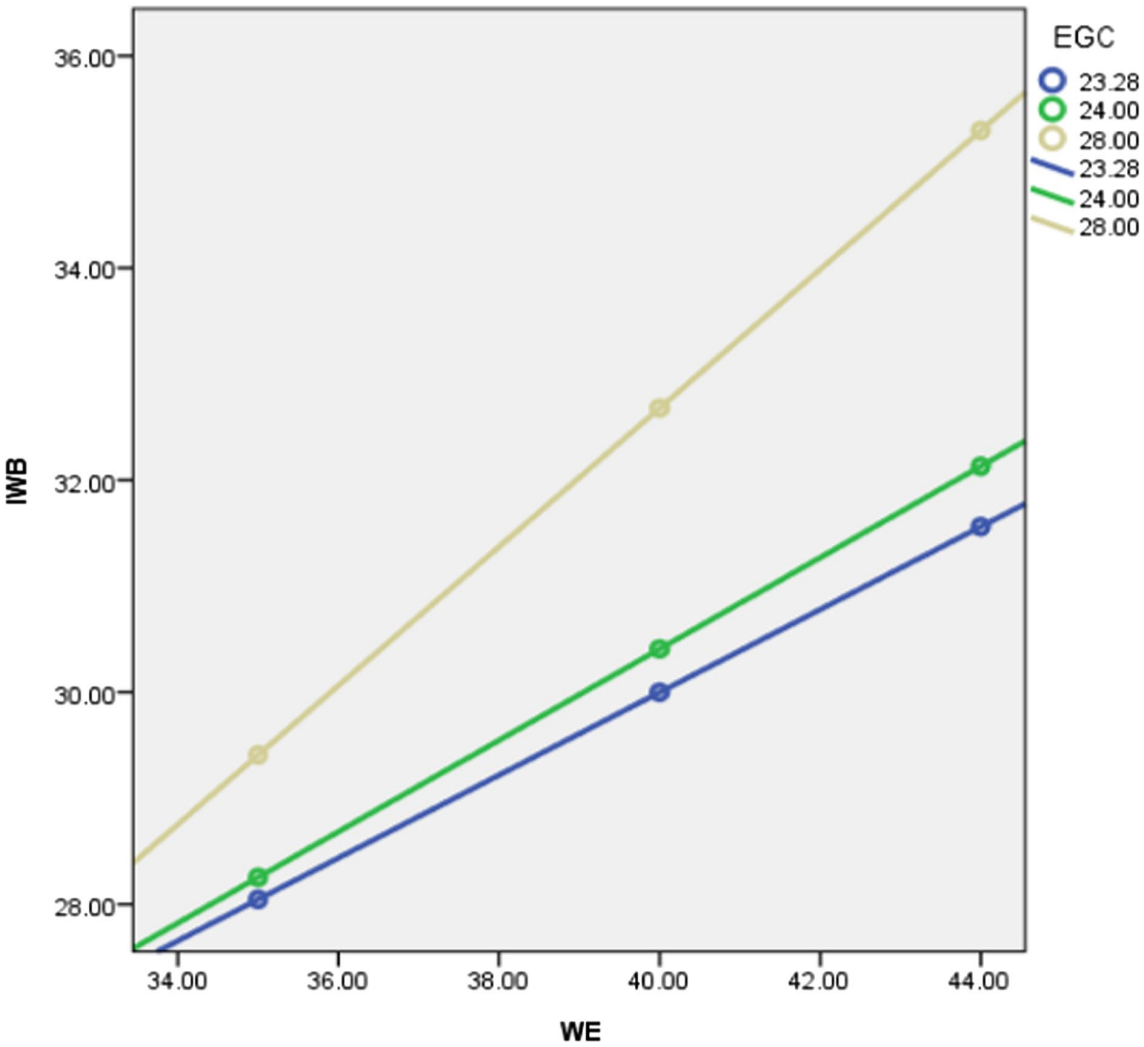
Interaction plot of innovative work behavior as a function of work engagement for low (−1SD), average, and high (+1SD) levels of employee growth climate.

Table 3 shows the result of the moderating effect of employee growth climate on the relationship between work engagement and innovative work behavior [*b* = 0.06, *t*(603) = 42.53, *p* = 0.00]. The result shows that employee growth climate significantly moderates the relationship between work engagement and innovative work behavior. Based on this analysis, hypothesis 2c confirmed. We then compute separate regression lines for high employee growth climate, as perceived by the employee (1 SD above the mean), average employee growth climate, and low employee growth climate (1 SD below the mean), and plotted this (see Figure 3).

Figure 3 displays the interaction between work engagement in the prediction of innovative work behavior. The figure illustrates that employee growth climate moderates the relationship between work engagement and innovative work behavior in such a way that work engagement is positively associated with innovative work behavior for high, average, and low employee growth climate. This finding supports hypothesis 2c. Hence, it is concluded that the relationship between work engagement and innovative work behavior is moderated by employee growth climate.

## Discussion

Moderated by employee growth climate, there were significant relations between work engagement and job outcomes among plantation workers in North Sumatra, Indonesia. Three types of individual-level job outcomes were included: intra-role behavior, employee learning, and innovative work behavior. The study found that the relations between work engagement-intra-role behavior and work engagement-innovative work behavior were moderated by employee growth climate (H1 and H3 were supported), however, the link was not significant between work engagement and employee learning (H3). The data also showed the fairly lower coefficient correlation of work engagement with employee learning compare to the other two job outcomes (Table 1), this might be the reason why employee growth climate does not moderate the relationship between work engagement and employee learning, as indicated by the insignificant interaction.

Furthermore, the perceived psychological safety in learning (Edmondson, 1999) and the national culture background (Hofstede and Hofstede, 2005) of the participants in this study may related to the insignificant result of this study. In this study, all participants are coming from Indonesian culture. As studied by Hofstede (Hofstede and Hofstede, 2005), Indonesia has a high power distance and collectivistic national culture. With high power distance national culture, Indonesian acknowledges unequal power which occurs from the hierarchical system including in leaders-employees relation. Employees, who are in the position of low status and low power, should seek consent from their leaders, who have authority, high status and high power, for whatever actions they wish to take. Employees feel ease when leaders support their actions. They need approval or support from the authorities in order to feel psychologically safe to transfer or share knowledge. Thus, despite learning informally among peer group, employees that originating from high power distance counties prefer to learn from credible learning sources with relevant learning protocols such as from their leaders of experts rather than self-directed learning (Kim and McLean, 2014) Moreover, employees from high power distance cultures see performance feedback as a command instead of suggestion (Kim and McLean, 2014). They are not allowed to question their leaders or authorities, which is a far from the performance feedback concept as a method of learning (Kim and McLean, 2014). In sum, the situated power distance environment which derived from the national culture might hinder employees’ self-initiative for learning or sharing knowledge among their peers as they may feel unsafe psychologically to do so unless the leaders grant it.

From the scale validation procedure, two items were removed from the employee climate growth measurement tool (“At my workplace, it does not matter to make mistakes because it contributes to learning”; “At my workplace, it is not necessary to work according to the book”). Both items are also related to psychological safety. Psychological safety must be met if employees are expected to be willing to take risks in making mistakes during their learning process. Individuals who engage in learning behaviors such as asking for help, seeking feedback, or speaking out about mistakes or faulty assumptions face an increased risk of being judged by others in a position to provide that support (Lee et al., 2003, 2004). Aforementioned, Indonesian employees, influenced by culture, are avoiding risks since they will follow the authorities, such as “the book” and support from the leaders. Employees from collectivistic cultures prefer harmony in their group/organization and they choose safety and security by avoiding to take risks that induce disharmony in group mainstream opinion (Hayes, 2017). Again, employees do not feel safe to initiate learning at work (for example sharing knowledge) which may against the authorities (less risk-taking). In a study, Chinese employees (who share high power distance national cultures with Indonesia) were found to be more hesitant to share knowledge because they were afraid of being punished (Kim and McLean, 2014).

Psychological safety could affect behavioral outcomes such as the team’s creativity (Madjar et al., 2002) and both individual learning (Carmeli et al., 2009; Carmeli and Gittell, 2009), and team learning (Edmondson, 1999; Wong et al., 2010). However, we might say, the participants of this study that are Indonesian employees perceive psychological safety slightly differently due to cultural influence which affect the way they learn at workplace. With high power distance and collectivist national culture, the plantation workers may prefer a more structured learning environment and the learning activities initiate by the authorities (the leaders). Thus, future research should consider to include the perceived psychological safety in relation with employees’ learning and their cultural background. Cross-cultural research is necessary to justify whether or not culturally diverse employees yield different results in employee learning, especially to compare and contrast between samples from high/low power distance national culture countries. Furthermore, we believe that the high power distance and collectivistic national cultures influence how employee growth climate is translated in these Indonesian workers, particularly when it comes to performing workplace learning. As a result, we recommend that leaders clearly communicate the anticipated behaviors from employees, such as employee learning, and provide full support for them. A formal and a more structured learning are also more preferable for Indonesian workers. They require authoritative guidance in order to voice their individual opinion or knowledge in a systematic manner rather than confronting the group opinion or knowledge informally. In sum, practically, we recommend that plantation management implement a formal learning environment, and leaders directly guide and direct the learning process, as in that way plantation workers feel safer to express and share their ideas because they were supported by authorities. More detailed rules or codes of conduct, such as dos and don’ts at work, should be defined in order to foster learning as a culture.

### Limitation and future research direction

There are some drawbacks to this study. First, we put our theoretical model to the test using only one source of data — employees – and only one method, a self-report survey. Although Spector argues that the problem of common method variance (CMV), a bias arising when both predictors and predicted variables stem from the same source, is overstated, we encourage future researchers to use the multisource and mixed methods in collecting the data (Spector, 2006). For instance, by using objective performance indicators, such as critical incidents or behavioral checklist. Next, the current study relied on a specific sample collected from one Indonesian holding company in the agricultural industry, which might raise a generalizability concern. Though data from one organization is better for capturing organizational specificity, caution is warranted when our results are applied to other organizations. The results of this study might be suitable to apply for equivalent companies’ and employees’ characteristics (such as national cultures). Further research is needed to replicate our research model in different settings in terms of companies, industries, and employees’ characteristics. We call for a future cross-cultural research to compare and contrast participants from different national cultures’ background. Lastly, this study incorporated a cross-sectional research design and it is recommended that in the future research, researchers adopt a longitudinal research design.

## Conclusion

The relationship between work engagement and job outcomes, again, has been proven in this study. This empirical evidence supports the need for organizations to nurture their employees’ work engagement. To do so, human resources management could measure the level of employees’ work engagement as part of their periodic performance management program since it predicts positive job outcomes in the organization. Furthermore, the key role of employee growth climate in strengthening the relationship between work engagement and job outcomes is supported by this study. Organizations can promote this specific type of climate through socialization programs. Furthermore, management should allow employees to exercise their knowledge and skills by learning and facilitating their innovative work behavior. To be specific, management should establish the clear the boundary on which area employees are allowed to make mistakes and set experiment, and to create a new-more efficient ways of working.

## Data availability statement

The raw data supporting the conclusions of this article will be made available by the authors, without undue reservation.

## Ethics statement

Ethical review and approval was not required for the study on human participants in accordance with the local legislation and institutional requirements. The patients/participants provided their written informed consent to participate in this study.

## Author contributions

VR and WS were responsible for the research design. VR, RF, and DN were responsible for data collection and writing the article. VR and DN were responsible for data analysis. WS was responsible for investigating the result and improving quality of the article. All authors contributed to the article and approved the submitted version.

## Funding

This study was supported by the World Class University (WCU) Grant 2021 Universitas Sumatera Utara.

## Conflict of interest

The authors declare that the research was conducted in the absence of any commercial or financial relationships that could be construed as a potential conflict of interest.

## Publisher’s note

All claims expressed in this article are solely those of the authors and do not necessarily represent those of their affiliated organizations, or those of the publisher, the editors and the reviewers. Any product that may be evaluated in this article, or claim that may be made by its manufacturer, is not guaranteed or endorsed by the publisher.
